# Prevalence and determinants of intravenous admixture preparation errors: A prospective observational study in a university hospital

**DOI:** 10.1007/s11096-021-01310-6

**Published:** 2021-08-07

**Authors:** Janique G. Jessurun, Nicole G. M. Hunfeld, Joost van Rosmalen, Monique van Dijk, Patricia M. L. A. van den Bemt

**Affiliations:** 1 Department of Hospital Pharmacy, Erasmus MC, University Medical Center Rotterdam, Rotterdam, The Netherlands; 2 Department of Intensive Care, Erasmus MC, University Medical Center Rotterdam, Rotterdam, The Netherlands; 3 Department of Biostatistics, Erasmus MC, University Medical Center Rotterdam, Rotterdam, The Netherlands; 4 Department of Epidemiology, Erasmus MC, University Medical Center Rotterdam, Rotterdam, The Netherlands; 5 Department of Internal Medicine, Section of Nursing Science, Erasmus MC, University Medical Center Rotterdam, Rotterdam, The Netherlands; 6 Department of Clinical Pharmacy and Pharmacology, University Medical Center Groningen, Groningen, The Netherlands

**Keywords:** Admixture, Determinant, Medication errors, Medication preparation, Medication safety, Risk factor

## Abstract

*Background* Intravenous admixture preparation errors (IAPEs) may lead to patient harm. Insight into the prevalence as well as the determinants associated with these IAPEs is needed to elicit preventive measures. *Aim* The primary aim of this study was to assess the prevalence of IAPEs. Secondary aims were to identify the type, severity, and determinants of IAPEs. *Method* A prospective observational study was performed in a Dutch university hospital. IAPE data were collected by disguised observation. The primary outcome was the proportion of admixtures with one or more IAPEs. Descriptive statistics were used for the prevalence, type, and severity of IAPEs. Mixed-effects logistic regression analyses were used to estimate the determinants of IAPEs.* Results* A total of 533 IAPEs occurred in 367 of 614 admixtures (59.8%) prepared by nursing staff. The most prevalent errors were wrong preparation technique (n = 257) and wrong volume of infusion fluid (n = 107). Fifty-nine IAPEs (11.1%) were potentially harmful. The following variables were associated with IAPEs: multistep versus single-step preparations (adjusted odds ratio [OR_adj_] 4.08, 95% confidence interval [CI] 2.27–7.35); interruption versus no interruption (OR_adj_ 2.32, CI 1.13–4.74); weekend versus weekdays (OR_adj_ 2.12, CI 1.14–3.95); time window 2 p.m.-6 p.m. versus 7 a.m.-10 a.m. (OR_adj_ 3.38, CI 1.60–7.15); and paediatric versus adult wards (OR_adj_ 0.14, CI 0.06–0.37).* Conclusion* IAPEs, including harmful IAPEs, occurred frequently. The determinants associated with IAPEs point to factors associated with preparation complexity and working conditions. Strategies to reduce the occurrence of IAPEs and therefore patient harm should target the identified determinants.

## Impacts on practice

- The high prevalence of intravenous admixture preparation errors found in this study emphasises the need for hospitals to develop strategies to prevent these errors.
- Multistep preparations, interruptions, preparation during the weekend or between 2 p.m. and 6 p.m., and adult wards were significantly associated with an increased probability of intravenous admixture preparation errors.
- Beneficial strategies may include increased use of ready-to-administer medication, support from pharmacy staff, educational programmes, and interruption management.
- Further studies should focus on the underlying causes of these errors to fully understand contributing factors, which subsequently may lead to tailored and effective preventive strategies.

## Introduction

Medication errors occur frequently and are associated with increased patient morbidity and mortality [1–5]. Especially intravenous drug therapy, including preparation of intravenous admixtures, poses an increased risk of medication errors and patient harm [3, 6, 7]. Preparation of intravenous medication is complex and error-prone due to the multistep nature of the task [6]. Negative effects after administration of erroneously prepared intravenous admixtures are more difficult to mitigate because of complete and immediate bioavailability. Systematic reviews showed that the rates of intravenous admixture preparation errors (IAPEs) varied substantially across studies, but high rates were quite common [4, 7–9]. For instance, a systematic review of Hedlund et al. reported wrong dose rates ranging from 0% to 32.6% and wrong concentration error rates from 0.3% to 88.6% [8]. The rates of harmful errors varied extensively between studies, but rates as high as 64% have been reported [8].

Several strategies have been explored to reduce the number of IAPEs [8, 10, 11], such as implementing pharmacy-based centralised intravenous admixture services (CIVAS) [11–13] or automated preparation systems [11, 14, 15]. These strategies might be cost-effective by preventing adverse drug events [16], which are associated with substantial costs, ranging from approximately €1000 to €7000 per event [17]. However, many of these strategies are associated with substantial direct investment costs and are therefore not feasible in all settings.

An alternative approach to identify preventive strategies is to gain insight into the determinants that are associated with IAPEs. Most studies that have been performed on determinants of IAPEs [18–25] have collected data retrospectively [23, 24], examined a limited number of determinant types [19, 23] or IAPE types [20], examined IAPEs as part of a composite endpoint with medication administration errors [18, 21, 22, 25], or included specific units such as cytotoxic preparation, intensive care, or paediatric units [21–25]. Consequently, contributing determinants vary substantially across studies, but encompass determinants such as preparation complexity, nursing staff experience, medication class, and workload. In addition, qualitative studies that have focused on causes of medication administration errors, which include preparation errors, report numerous potential causes related to personal factors (e.g. fatigue and stress), knowledge-based factors (e.g. lack of knowledge of protocols or use of technology), and contextual factors (e.g. heavy workload, time pressures, interruptions, and lack of training) [26–30]. These findings suggest a need for a multifaceted approach to reduce the number of IAPEs.

Studies that quantify determinants of IAPEs are necessary to elicit preventive strategies, but quantitative studies in general clinical wards are scarce.

### Aim

We performed an observational study in five general clinical wards aimed to assess the prevalence, type, and severity of IAPEs as well as the determinants associated with the occurrence of IAPEs.

### Ethics approval

The Medical Ethics Review Committee of Erasmus MC waived approval for this study (reference number MEC-2018–1170) in accordance with the Dutch Medical Research involving human subjects Act. Before study enrolment, nursing staff were informed that data will be collected for research purposes, aiming to optimise the medication distribution process. Nursing staff gave verbal consent for participation in this study. Data were handled according to the Dutch General Data Protection Regulation.

## Method

### Study design

A prospective observational study was conducted in five clinical wards (haematology, internal oncology, neurosurgery, and two paediatric wards) in Erasmus MC, University Medical Center Rotterdam in the Netherlands.

### Study setting

Study enrolment took place from January 9, 2018 until March 21, 2018. Nursing staff used workbenches in the medication rooms on the clinical wards to prepare intravenous admixtures. Medication preparation instructions were available in the electronic handbook and medication prescriptions in the electronic medical record (EMR) system HiX® version 6.1 (Chipsoft B.V.; Amsterdam, the Netherlands) and in the computerised physician order entry (CPOE) system Practocol® version 2.0.8.2 (Practocol B.V.; Rotterdam, the Netherlands) for medication in chemotherapy protocols. In adult clinical wards, intravenous admixtures were prepared by nursing staff. In paediatric wards, admixtures were prepared by non-nursing personnel (e.g. medical students) during office hours from Monday through Sunday. They carried out medication tasks in the medication room, including preparation of planned medication, under supervision. Unplanned and urgently needed intravenous admixtures were prepared by nursing staff.

### Inclusion and exclusion criteria

All types of intravenous admixture preparations that were performed by nursing staff for administration to inpatients were included in this study. Admixture preparations that were not finished during the observation or could not be linked to a specific patient or medication were excluded. Preparation of parenteral nutrition and cytotoxic medication were not within the scope of this study, because preparation took place in the hospital pharmacy.

### Definitions and classification of IAPE

An IAPE was defined as any error in the preparation of an intravenous admixture, i.e. a deviation from the medication order, a deviation from the local electronic admixture preparation instructions, or a deviation from the medication information sheet provided by the manufacturer in case local protocols were not available [7, 31]. Procedural errors (e.g. hygiene and labelling errors) were not within the scope of this study.

The IAPEs were classified into the following types [31]: 1. wrong drug, 2. wrong dose, 3. wrong solvent or diluent, 4. wrong volume of solvent or diluent, 5. wrong infusion fluid, 6. wrong volume of infusion fluid, 7. wrong preparation technique (e.g. incomplete mixing), and 8. other. For the categories wrong dose and wrong volume, a deviation of more than 10% was considered incorrect, because this deviation is expected to be within the limits of individual variation and widely accepted [32]. The potential severity of IAPEs was classified according to the National Coordinating Council for Medication Error Reporting and Prevention (NCC MERP) index [33].

### Study outcomes

The primary outcome was the proportion of intravenous admixtures with one or more IAPEs, estimated by dividing the number of intravenous admixtures with one or more IAPEs by the total number of intravenous admixtures included. Secondary outcomes were the frequencies of the type and severity of IAPEs as well as the association between determinants and the occurrence of one or more IAPEs. Determinants were selected based on proposed associations [18–26, 34] and on theoretical assumptions. The following potential determinants were considered: preparation complexity; pharmaceutical form; medication class; interruptions; day of the week; time window; clinical ward type; and nursing staff gender, age, degree type, educational level, and experience since first nursing-diploma registration.

### Data collection

Data on intravenous admixture preparation were collected by disguised observation [35–37], meaning that the staff members were not informed about the detailed purpose of the study, to prevent them from altering their behaviour (i.e. the Hawthorne effect). Trained observers, mostly students with a medical or pharmaceutical background, accompanied the nursing staff in daily clinical practice to observe and document every dose preparation on standard data collection forms. Observers asked nurses for verbal consent before initiating an observation. Observation rounds were planned in periods of 1–3 weeks for each clinical ward. Observers were instructed to only intervene in case of a serious error [36]. Observation data were compared with medication orders and protocols after the observation and not during observation, which is in accordance with the gold standard of medication error detection methods [36]. Thus only very obvious serious errors would be likely to be intervened upon.

The observation forms were independently reviewed by a pharmacist (JJ) and hospital pharmacist (NH) to determine the presence, type, and severity of IAPEs; disagreements between assessments were resolved by consensus.

The determinants preparation complexity (single step, multistep), medication class by Anatomical Therapeutic Chemical (ATC) class [38], and day of the week were assessed by one pharmacist (JJ). For the complexity assessment, admixtures were defined as multistep if at least one of the following criteria was met: (1) preparation using an injection powder, (2) preparation with three or more medication vials, (3) syringe preparation after diluting injection liquids or infusion liquids, or (4) preparation of individual dosages requiring complex calculations. Nursing staff were asked (by e-mail and/or in person) to fill in a questionnaire to collect their background characteristics in terms of gender, age, degree type, educational level, and experience since first nursing-diploma registration, after completion of the observation periods in a particular unit. One pharmacist (JJ) collected data on patient characteristics, i.e. gender, birth date, and number of prescribed medications per day, from the EMR and CPOE. Data on other determinants, i.e. pharmaceutical form, number of interruptions, time window, and clinical ward type, were collected during observation.

Collected data were entered in OpenClinica® version 2.1 (OpenClinica LLC; Waltham, Massachusetts, United States).

### Sample size calculation

Assuming an IAPE rate of 15% [1, 3, 4, 8, 31, 39–44] and using the rule of thumb that one predictive variable can be studied for every 10 events [45], a sample size of 800 intravenous admixtures would be required for examining 12 variables. Seventy-five observation rounds were planned beforehand, based on the expected number of intravenous admixtures observed per observation round.

### Data analysis

Descriptive statistics were used to analyse the prevalence, type, and severity of IAPEs. Univariable and multivariable mixed-effects logistic regression analyses (i.e. generalised linear mixed models) were used to determine the association between potential determinants and the occurrence of IAPEs. These models account for within-subject correlations due to repeated measurements by staff member and patient. To adjust for substantial data dependence, only the first admixture was included for matched admixtures, i.e. admixtures with the following five identical characteristics: staff member, patient, medication name, time window, and date of admixture preparation. To take into account data dependence due to multicollinearity and to maintain sufficient statistical power despite the fact that the sample size was lower than planned, we made a selection of determinants and we combined some categories, based on theoretical associations and available literature [18–26, 30, 34]. The following variables were examined in the univariable and multivariable mixed-effects logistic regression analyses: preparation complexity (categorised; single step, multistep); interruptions (categorised; yes, no); day of the week (categorised; weekdays, weekend); time window (categorised; 7 a.m.–10 a.m., 10 a.m.–2 p.m., 2 p.m.–6 p.m., 6 p.m–7 a.m.); and clinical ward type (categorised; adult wards, paediatric wards). Nursing staff characteristics were excluded from further analysis because these data were only available for 35 out of 109 observed staff members. Pharmaceutical form and medication class were excluded because of multicollinearity with preparation complexity. All injection powders and almost all anti-infective medications require multistep preparations. Therefore preparation complexity overlaps with these multistep prepared medications, which induces multicollinearity. To account for repeated measurements and the within-subject correlations, we included two random effects, i.e. a random intercept by staff member and a random intercept by patient, which led to a model with crossed random effects. For the multivariable analysis, a complete case analysis was performed. Adjusted odds ratios with 95% confidence intervals were used for the results of the mixed-effects logistic regression analyses. These odds ratios should be interpreted conditionally on the random effects, i.e. they represent a comparison of two observations of the same staff member and patient.

For all statistical analyses a two-sided significance level of 0.05 was chosen. Data analyses were performed with R Statistics® version 4.0.2. (The R Foundation; Vienna, Austria) for the mixed-effects logistic regression analyses and with SPSS Statistics® version 25 (IBM Corporation, Armonk, New York, United States) for other analyses.

## Results

A total of 621 intravenous admixture preparations were observed. Seven admixture preparations were excluded because of missing data (n = 6, patient identifier; n = 1, medication name). Hundred and nine nursing staff members were observed during the preparation of intravenous admixtures for 117 patients. The characteristics of included admixtures, staff members, and patients are shown in Table 1. Observers did not intervene in any intravenous admixture preparation.

**Table 1:** Characteristics of included intravenous admixtures, staff members, and patients

Intravenous admixtures—*n*	614
*Characteristics*	
*Medication characteristics*	
Preparation complexity—n (%)	
Multistep	477 (77.7)
Pharmaceutical form of medication vial—n (%)	
Injection powder	262 (42.7)
Injection liquid	233 (37.9)
Infusion	119 (19.4)
Medication class (ATC code)—n (%)	
Alimentary tract and metabolism (A)	86 (14.0)
Blood and blood forming organs (B)	38 (6.2)
Cardiovascular system (C)	50 (8.1)
Systemic hormonal preparations (H)	11 (1.8)
Anti-infectives for systemic use (J)	268 (43.6)
Antineoplastic and immunomodulating agents (L)	35 (5.7)
Nervous system (N)	103 (16.8)
Miscellaneous	23 (3.7)
*Environmental characteristics*	
Interruptions—n (%)	
Yes	102 (16.6)
*Ward characteristics*	
Clinical ward type—n (%)	
Adult wards	
Haematology	316 (51.5)
Internal oncology	79 (12.9)
Neurosurgery	24 (3.9)
Paediatric wards	
Paediatric ward one	56 (9.1)
Paediatric ward two	139 (22.6)
*Time characteristics*	
Day of the week—n (%)	
Monday	87 (14.2)
Tuesday	97 (15.8)
Wednesday	55 (9.0)
Thursday	85 (13.8)
Friday	115 (18.7)
Saturday	59 (9.6)
Sunday	116 (18.9)
Time window ^a^—n (%)	
7 a.m–10 a.m	117 (19.1)
10 a.m–2 p.m	183 (29.8)
2 p.m–6 p.m	224 (36.5)
6 p.m–7 a.m	88 (14.3)
*Staff members *^*b*^	
Observed staff members—n	109
Staff members, personal data available—n (%)	35 (32.1)
Male—n (%)	3 (8.6)
Age—median (IQR)	30 (26–45)
Degree type—n (%)	
Nurse	12 (34.3)
Specialised nurse	17 (48.6)
Student nurse	3 (8.6)
Other	3 (8.6)
Educational level—n (%)	
Secondary vocational education	14 (40.0)
Higher professional education	19 (54.3)
University education	2 (5.7)
Experience since nursing diploma—n (%)	
0 to 1 year	1 (2.9)
1 to 5 years	7 (20.0)
More than 5 years	22 (62.9)
Not applicable	5 (14.3)
*Patients*	
Patients—n	117
Male—n (%)	69 (59.0)
Age—median (IQR)	
Adult wards	60 (51–67)
Paediatric wards	6 (1–14)
Prescribed medications per day—median (IQR)	13 (9–16)

ATC, Anatomical Therapeutic Chemical; IQR, interquartile range

^a^Missing time window: 2 admixtures

^b^Missing nursing staff identifier: 14 admixtures

Table 2 shows the prevalence, type, and potential severity of IAPEs stratified by clinical ward type. One or more IAPEs occurred in 367 of 614 admixtures (59.8%) and in 323 of 490 admixtures (65.9%), respectively, before and after excluding matched admixtures.

**Table 2:** Prevalence, type, and severity of intravenous admixture preparation errors (IAPEs) stratified by clinical ward type

	Haematology	Internal oncology	Neurosurgery	Paediatric ward one	Paediatric ward two	Total
Intravenous admixtures—n	316	79	24	56	139	614
*Prevalence of IAPEs—n (%)*						
Admixtures with one or more IAPEs	236 (74.7)	49 (62.0)	18 (75.0)	20 (35.7)	44 (31.7)	367 (59.8)
IAPEs—n	329	70	24	39	71	533
*Type of IAPEs—n (%)*						
Wrong drug	1 (0.3)	4 (5.1)	0	0	1 (0.7)	6 (1.0)
Wrong dose	18 (5.7)	0	1 (4.2)	5 (8.9)	3 (2.2)	27 (4.4)
Wrong solvent or diluent	87 (27.5)	0	0	0	0	87 (14.2)
Wrong volume of solvent or diluent	9 (2.8)	7 (8.9)	2 (8.3)	0	4 (2.9)	22 (3.6)
Wrong infusion fluid	3 (0.9)	2 (2.5)	4 (16.7)	7 (12.5)	9 (6.5)	25 (4.1)
Wrong volume of infusion fluid	66 (20.9)	13 (16.5)	1 (4.2)	7 (12.5)	20 (14.4)	107 (17.4)
Wrong preparation technique	144 (45.6)	44 (55.7)	16 (66.7)	19 (33.9)	34(24.5)	257 (41.9)
Incomplete mixing	141	42	16	12	29	240
Other	1 (0.3)	0	0	1 (1.8)	0	2 (0.3)
*Severity of IAPEs* ^*a*^*—n (%)*						
Error, no harm						
C	225 (71.2)	64 (81.0)	23 (95.8)	34 (60.7)	58 (41.7)	404 (65.8)
D	58 (18.4)	0	1 (4.2)	1 (1.8)	10 (7.2)	70 (11.4)
Error, harm						
E	42 (13.3)	3 (3.8)	0	3 (5.4)	3 (2.2)	51 (8.3)
F	3 (0.9)	3 (3.8)	0	1 (1.8)	0	7 (1.1)
H	1 (0.3)	0	0	0	0	1 (0.2)

^a^NCC MERP classification: no error (category A); error, no harm (category B to D); error, harm (category E to H); and error, death (category I). C: an error occurred that reached the patient but did not cause patient harm; D: an error occurred that reached the patient and required monitoring to confirm that it resulted in no harm to the patient and/or required intervention to preclude harm; E: an error occurred that may have contributed to or resulted in temporary harm to the patient and required intervention; F: an error occurred that may have contributed to or resulted in temporary harm to the patient and required initial or prolonged hospitalisation; H: an error occurred that required intervention necessary to sustain life

The associations between determinants and the occurrence of IAPEs are shown in Table 3. The following variables were significantly associated with an increased probability of IAPEs in the multivariable analysis: multistep preparations versus single-step preparations (adjusted odds ratio [OR_adj_] 4.08, 95% confidence interval [CI] 2.27–7.35); interruptions versus no interruption (OR_adj_ 2.32, CI 1.13–4.74); weekend versus weekdays (OR_adj_ 2.12, CI 1.14–3.95); and time window 2 p.m.-6 p.m. versus 7 a.m.-10 a.m. (OR_adj_ 3.38, CI 1.60–7.15). Paediatric wards were associated with a lower probability of IAPEs versus adult wards (OR_adj_ 0.14, CI 0.06–0.37). The IAPE prevalence stratified by medication class is shown in Table 4.

**Table 3:** Association between determinants and the occurrence of intravenous admixture preparation errors (IAPEs) in clinical wards

Determinants	Mixed-effects logistic regression analysis ^a b^		
	Univariable analysis		Multivariable analysis n = 475
	n	Odds ratio (95% confidence interval)	Adjusted odds ratio (95% confidence interval)
*Medication characteristics*			
Preparation complexity	478		
Single step		Reference	Reference
Multistep		3.45 (1.96–6.06)*	4.08 (2.27–7.35)*
*Environmental characteristics*			
Interruptions	477		
No		Reference	Reference
Yes		1.96 (1.00–3.83)*	2.32 (1.13–4.74)*
*Time characteristics*			
Day of the week	478		
Weekday		Reference	Reference
Weekend		1.85 (1.02–3.36)*	2.12 (1.14–3.95)*
Time window	476		
7 a.m.–10 a.m		Reference	Reference
10 a.m.–2 p.m		2.53 (1.20–5.33)*	2.00 (0.97–4.13)
2 p.m.–6 p.m		3.54 (1.62–7.77)*	3.38 (1.60–7.15)*
6 p.m.–7 a.m		1.46 (0.63–3.37)	1.31 (0.59–2.94)
*Ward characteristics*			
Clinical ward type	478		
Adult wards		Reference	Reference
Paediatric wards		0.36 (0.16–0.83)*	0.14 (0.06–0.37)*

^a^Only the first admixture was included for matched admixtures, i.e. admixtures with the following identical characteristics: staff member, patient, medication name, time window, and date of admixture preparation

^b^Mixed-effects logistic regression analysis was used to account for within-subject correlations due to repeated measurements by staff member and patient

^*^Statistically significant (*p* < 0.05)

**Table 4:** Prevalence of intravenous admixture preparation errors (IAPEs) stratified by medication class

	Admixtures with one or more IAPEs—*n/N (%)*
*Medication class (ATC code)*	
Alimentary tract and metabolism (A)	44/86 (51.2)
Blood and blood forming organs (B)	24/38 (63.2)
Cardiovascular system (C)	7/50 (14.0)
Systemic hormonal preparations (H)	10/11 (90.9)
Anti-infectives for systemic use (J)	228/268 (85.1)
Antineoplastic and immunomodulating agents (L)	28/35 (80.0)
Nervous system (N)	12/103 (11.7)
Miscellaneous	14/23 (60.9)

ATC Anatomical therapeutic chemical

## Discussion

In this study, IAPEs, including potentially harmful IAPEs, were frequently observed, that is in almost 6 out of 10 admixtures. Multistep preparations, interruptions, preparation during the weekend or between 2 p.m. and 6 p.m., and adult wards were significantly associated with an increased probability of IAPEs.

Previously reported IAPE rates varied substantially, from 0 to 90%, which may be explained by clinical and methodological heterogeneity (e.g. differences in IAPE types, data collection methods, and settings) [7, 8]. Therefore, the IAPE rate we established is difficult to compare to rates of previous studies. The most frequently observed IAPE types were wrong preparation technique, particularly incomplete mixing, and wrong volume of infusion fluid. Of all errors, 11.1% were potentially harmful, which is a lower percentage than in most previous studies [8]. This may be partially explained by the fact that these studies did not examine the frequently occurring IAPE type incomplete mixing, which is frequently considered not harmful, because, for many intravenous admixtures in infusion bags, spontaneous mixing is expected to occur during normal handling [46]. This is not the case for all medications and all container types. For instance, concentrated electrolytes, such as potassium chloride, may have serious consequences [47–52].

As expected and in line with previous studies [18, 53], multistep preparations increased the risk of errors compared to single-step preparations. Every additional step introduces an additional opportunity for error [6], making multistep preparations error-prone.

Being interrupted during preparation also increased the risk of errors. Studies report interruptions to occur frequently in the medication administration and preparation process [54–57] and to be a significant cause of medication errors [26–30, 55, 57]. Interruptions require individuals to switch attention from one task to another, which may have negative impacts on their performance, as they have to regain the context of the original task after completing the interrupted task [58].

In addition, both the weekend and time window 2 p.m. to 6 p.m. were error-prone periods. Previous studies show inconsistent results on these time-related determinants [18, 19, 22, 25]. In the included wards, staffing patterns differed during the weekend compared to weekdays and, in general, the number of tasks is distributed unevenly during the day. Thus, the identified error-prone periods may be related to factors such as workload and vigour/fatigue, which have been widely suggested as potential causes of medication errors [26, 28–30]. Nurses have previously reported that intravenous medication tasks are frequently rushed, mainly before shift changes, lunch breaks, or between ward rounds in order to focus on other tasks or to reduce the workload for the next shift [28].

Preparation in paediatric wards was associated with a decreased probability of errors. One contributing factor is that many preparations (approximately 75%) in the paediatric wards did not include a mixing step, the most error-prone step in the preparation process, accounting for 45% of all errors. Another contributing factor could be the fact that these paediatric wards hired staff, such as nursing and medical students, specifically for medication tasks, including preparation of planned intravenous medication, but its contribution remains to be explored.

Thus, with regard to studied determinants, our study confirms most of the findings of previous studies [18–25]. To our knowledge, our study is the first prospective study that used direct observation, corrected for repeated measurements on nurse and patient level, and directly compared paediatric wards and adult wards.

A strength of this study is that we included admixture preparations intended for inpatients in five different clinical wards, which support the generalisability of the results of this study to similar hospitals. Another strength is that we used a robust method to determine the occurrence of IAPEs.

This study has some limitations. First, the disguised observation method is the gold standard to detect medication errors, but observer bias may have occurred [35, 37]. However, several measures have been taken to limit this observer bias, such as establishing simple (e.g. by using fixed fields) and thorough data collection forms to support observers as well as extensive training programmes for observers. The presence of observers may cause altered behaviour of the observed in a positive sense (Hawthorne effect), which may lead to an underestimation of the IAPE rate, but by not revealing the exact purpose of the observations, this effect is minimised. Second, the low response rate on the questionnaire to collect data on staff characteristics resulted in a poor description of nursing staff and impeded analyses of nursing staff related determinants. Finally, the observational study design precludes conclusions on causal associations.

This study showed that IAPEs, including potentially harmful IAPEs, are common in clinical practice, which merits implementation of effective systemic defences to improve patient safety. Previously investigated strategies to reduce the number of IAPEs include quite comprehensive and costly interventions such as automated preparation systems [11, 14, 15] and centralising intravenous admixture preparation to hospital pharmacies [11–13, 59]. Our study identified determinants that may be the focus of other interventions. The identified determinants point to factors related to the complexity of preparations and working conditions. Strategies may include increased use of ready-to-administer medication [10, 11, 16], support from pharmacy staff [11], focused educational programmes [60], or interruption management interventions [57, 60]. Especially increased used of ready-to-administer medication, pharmacy or industry manufactured, is very promising [10, 11, 16], as it has been shown to be cost-effective [16]. However, preparation on wards will still be needed, as not all medications are suitable to be produced in a ready-to administer form, for example because of stability issues. Therefore, it remains crucial for nursing staff to prepare intravenous admixtures regularly and to receive tailored education on this matter.

Future studies should focus on the effectiveness of preventive strategies [11] and on potential determinants insufficiently identified by our study (e.g. workload and nurse characteristics) as well as the underlying causes of IAPEs specifically.

## Conclusion

This study showed that IAPEs, including potentially harmful IAPEs, are prevalent in a hospital setting. We identified several determinants associated with the occurrence of these IAPEs. These determinants point to factors associated with preparation complexity as well as working conditions. Strategies to reduce the occurrence of IAPEs and therefore patient harm should target the identified determinants.
